# Computed tomography imaging-guided parasternal approach drainage for children with tension pneumomediastinum: a case series

**DOI:** 10.1186/s12887-023-04417-z

**Published:** 2023-11-22

**Authors:** Jun-Jie Hong, Song-Ming Hong, Xiu-Hua Chen, Si-Jia Zhou, Qiang Chen, Jin-Xi Huang

**Affiliations:** Department of Cardiothoracic Surgery, Fujian Children’s Hospital (Fujian Branch of Shanghai Children’s Medical Center), College of Clinical Medicine for Obstetrics & Gynecology and Pediatrics, Fuzhou, China

**Keywords:** Computed tomography guided, Parasternal approach drainage, Tension pneumomediastinum, Pigtail catheter, COVID-19, Children

## Abstract

**Purpose:**

Tension pneumomediastinum is a rare and dangerous complication in children that can be fatal, and timely detection and treatment are critical. The aim of this study was to evaluate the safety and feasibility of computed tomography (CT) imaging-guided parasternal approach drainage for tension pneumomediastinum in children.

**Methods:**

From June 2018 to February 2023, we consecutively enrolled 19 children with tension pneumomediastinum in our institution. A pigtail catheter was inserted into the anterior mediastinum by a CT imaging-guided parasternal approach. The catheter was connected to a negative-pressure water seal bottle to drain the pneumomediastinum. Clinical data and outcomes were summarized.

**Results:**

The mean age was 3.1 ± 3.4 years, the mean weight was 15 ± 9.1 kg, the mean procedure time was 11.8 ± 2.4 min, and the drainage time was 6.7 ± 3.4 days. No major complications were identified, such as haemothorax, catheter displacement, or mediastinal infection. Effective drainage was obtained in all patients as assessed by comparing images and ventilatory parameters, and no additional surgical treatment was needed. There was no recurrence during the follow-up, which was more than 2 months. In our data, two children with COVID-19 were discharged from the hospital after effective drainage and other clinical treatment.

**Conclusion:**

CT-guided parasternal approach drainage is safe, minimally invasive, and effective for children with tension pneumomediastinum.

## Introduction

Laennec [1] first described pneumomediastinum as the presence of free air in the mediastinum. Its clinical symptoms are cough, dyspnoea, chest pain, and acute respiratory dysfunction, which may occur in severe cases. Tension pneumomediastinum (TPM), one of its severe complications, has been reported in only a few cases [2]. Pneumomediastinum, associated with severe hypoxia, tachycardia, metabolic acidosis, and high ventilation pressures, is characterized by clinically significant tension in the mediastinum [3]. Chest radiography shows that the cardiac silhouette appears flattened, indicating cardiac compression caused by tension pneumomediastinum, called the earth-heart sign [4], which is a typical imaging manifestation of tension pneumomediastinum. In addition to a flattened anterior cardiac contour, computed tomography (CT) scans can sometimes also show compression of the aorta, vena cava, or tracheobronchial tree. If left untreated, it may develop into a life-threatening condition such as cardiac tamponade [5]. Traditional treatments include tracheotomy [2], sternotomy [6], and mediastinotomy [7]. New therapies include parasternal approach drainage [1, 8–10] and posterior mediastinal drainage [11]. We reviewed the clinical data of 19 children with tension pneumomediastinum who underwent CT imaging-guided parasternal approach drainage and summarized our experience.

### Patients and methods

The present study adhered to the tenets of the Declaration of Helsinki and was approved by the Ethics Committee of our hospital. Additionally, written informed consent was obtained from the parents of the patients.

### Patients

**Fig. 1:**
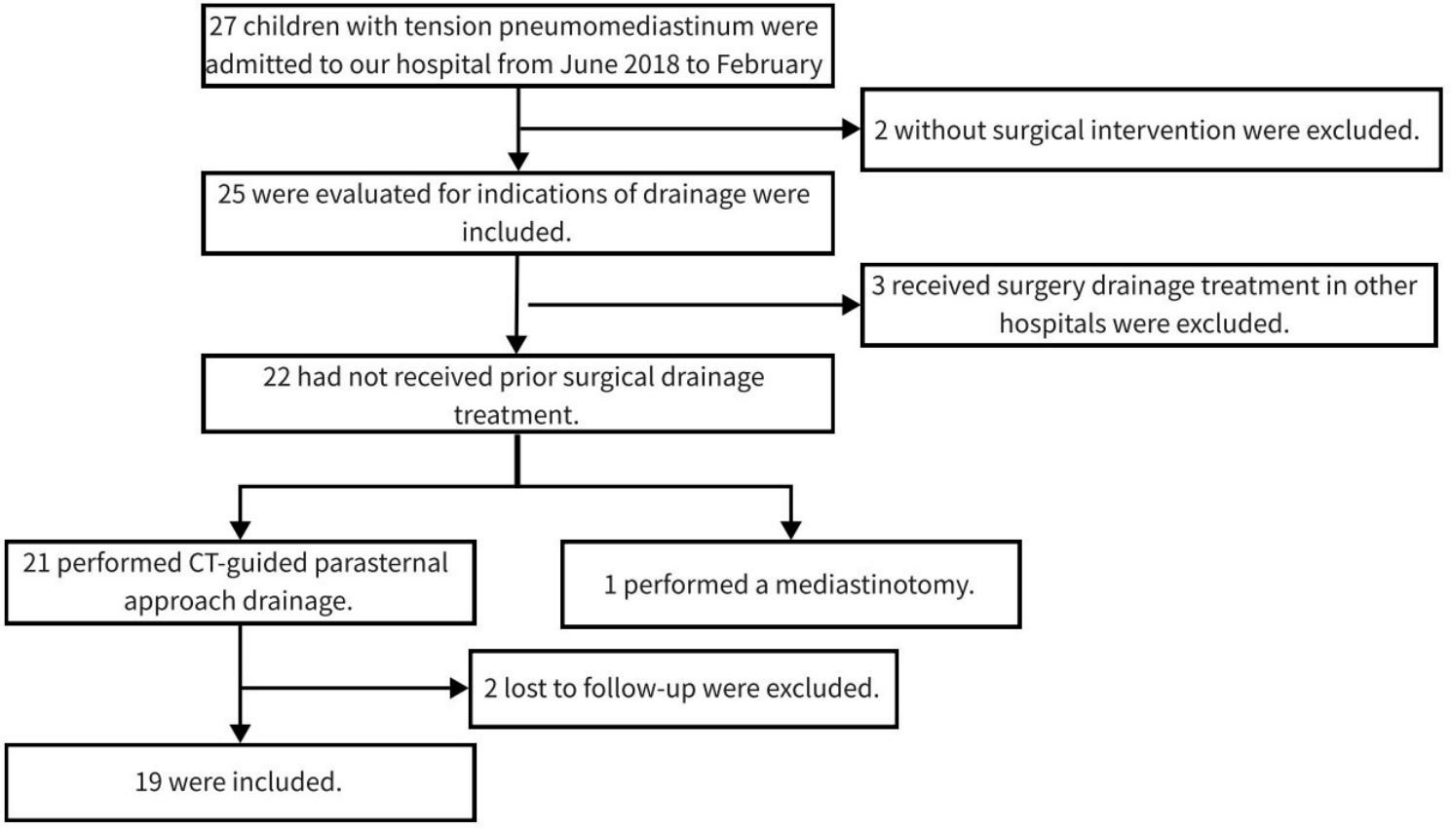
CONSORT flow diagram of participants

From June 2018 to February 2023, 27 children with tension pneumomediastinum were admitted to our hospital. The selection criteria were patients who accepted CT imaging-guided parasternal approach drainage. The exclusion criteria were as follows: (1) Patients received surgical drainage treatment in other hospitals. (2) Patients received traditional surgical approaches, including tracheotomy, sternotomy, and mediastinotomy. (3) Patients with incomplete clinical data and those lost to follow-up. Finally, 19 patients were included (Fig. 1). The indications for drainage were as follows [4]: (1) The patient needed mechanical ventilation due to acute respiratory deterioration and hemodynamic instability that was not improved after general treatment. (2) Imaging suggested significant compression of the heart, aorta, vena cava, or tracheobronchial tree.

### Surgical technique

**Fig. 2:**
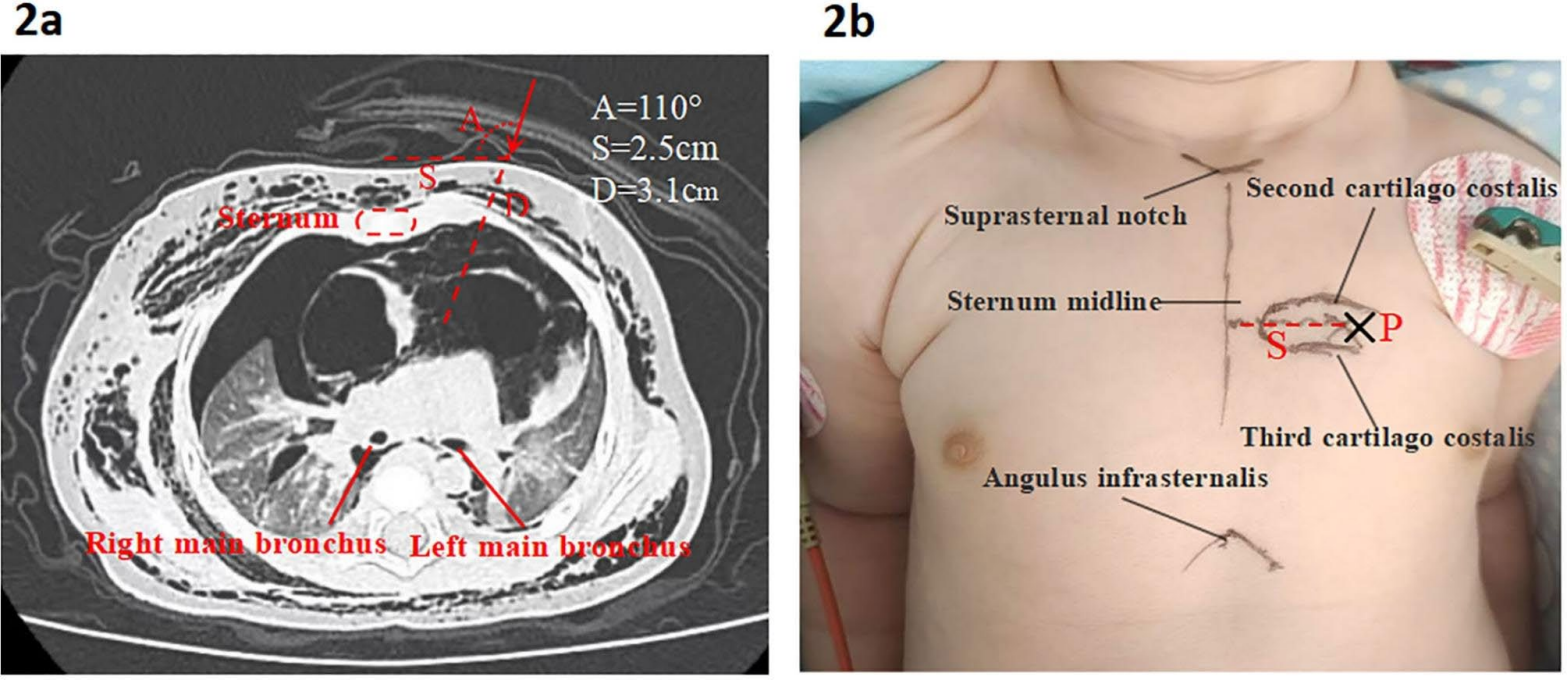
2**a**: Plan the puncture path using preoperative chest computed tomography. Extensive mediastinal tension emphysema compressing the mediastinal vessels and both lungs, the trachea is pushed posteriorly. Extensive lateral subcutaneous emphysema. 2**b**: Skin surface markers of the puncture site. A: The angle of needle insertion; D: The depth of pneumomediastinum; S: The spacing between the puncture site and the sternum midline. P: The puncture site

The same paediatric thoracic surgical treatment group performed all procedures. The CT machine type was RevolutionCT; the average radiation dose of a single chest CT scan was approximately 1 mSv, and the radiation time was approximately 3 s. The puncture site and path were planned according to CT imaging and 3D reconstruction, combined with bony markers. The depth (D) of pneumomediastinum, the spacing (S) between the puncture point and the midline of sternum, and the angle (A) of needle insertion were measured based on CT imaging, with the central emphysema area as the puncture site, and the puncture site (P) was marked on the skin surface (Fig. 2).

Sedation method: Midazolam was slowly infused intravenously at an initial dose of 0.05–0.1 mg/kg and then continuously infused intravenously at a dose of 1–2 µg/kg/min to maintain sedation. After sufficiently sedating the patient, 1% lidocaine was used for local anaesthesia. A modified Seldinger technique was used [12]. Mediastinal puncture was performed using a 20-gauge needle attached to a 5-mL syringe to avoid significant damage. While applying negative pressure, insert the needle along the selected puncture site along the upper margin of the rib. Stop inserting the needle when air or fluid is obtained. Fix the needle in position and remove the syringe. Insert the guidewire along the needle core until it is deep enough into the mediastinum. Insert the dilator over the guide wire and carefully twist it to dilate the skin, muscles, and pleura. Once dilation is complete, remove the dilator, keeping the guidewire in place. Thread the caudal fibre catheter over the guidewire. After successful catheterization, fix the catheter to the skin around the puncture site with sutures, and connect the catheter to a sealed negative pressure water seal bottle. For drain size, we prefer a small chest tube (8–14 Fr) recommended by the Danish Pulmonary Society (DLS) [13]. In general, 6–8 Fr pigtail catheters were used for patients weighing < 10 kg, and 10 Fr catheters were used for patients weighing > 10 kg and adjusted to the situation.

**Fig. 3:**
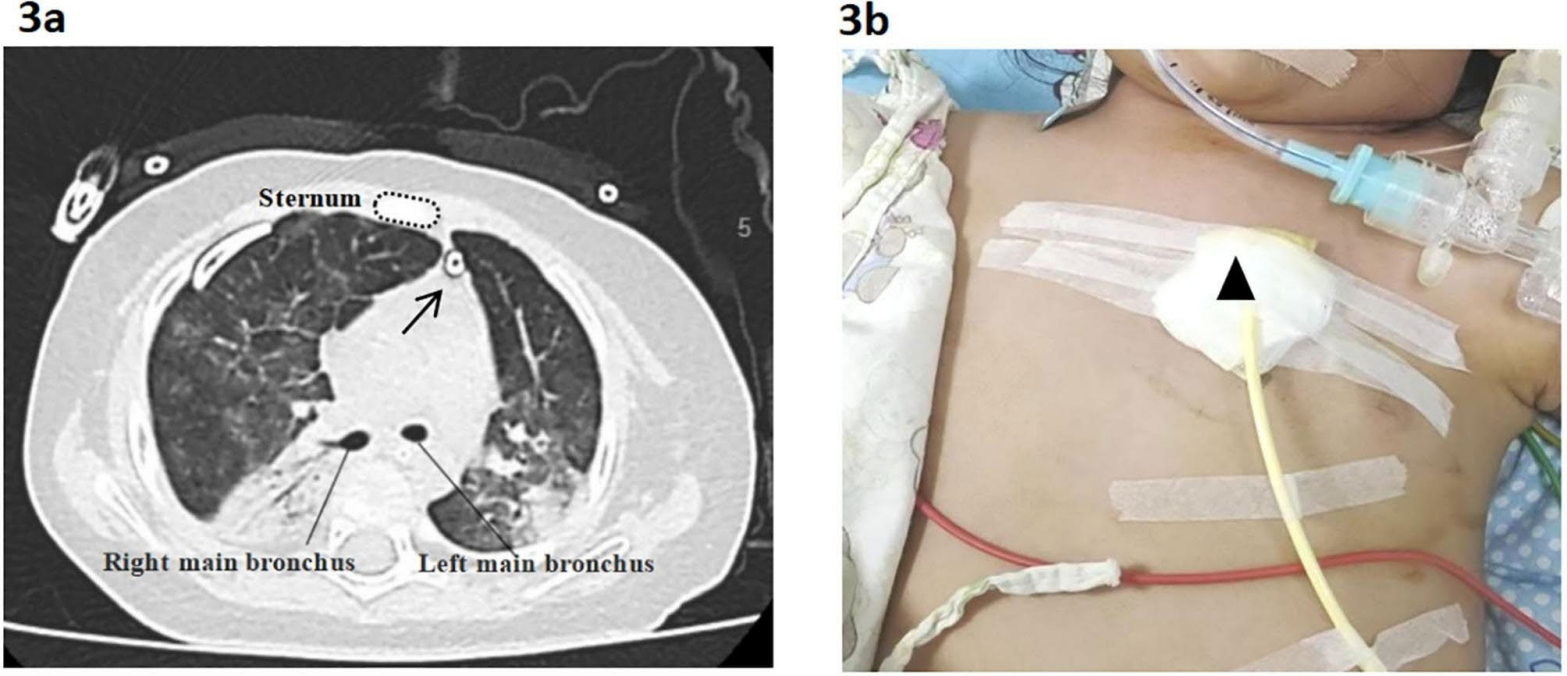
3**a**: Postoperative chest CT showed a satisfactory location of the pigtail catheter in the anterior mediastinum. (→). 3**b**: The place to insert the pigtail catheter.(▲)

Postoperative bedside X-ray was used to determine catheter location and evaluate efficacy. The patient’s radiation exposure did not increase. Three of them underwent chest CT because of pneumonia progression, and a satisfactory catheter location was seen from their CT images (Fig. 3). The response to treatment was measured by comparing images and ventilatory parameters before and after catheter drainage and by assessing each patient’s clinical outcome.

## Statistical analysis

Continuous data are presented as the mean ± standard deviation and range, and the categorical variables are presented as frequencies (%). Clinical parameters are shown in Table 1. SPSS (Windows version 26.0 IBM Co, Armonk, NY, USA) was used for all statistical analyses.

**Table 1:** Demographical and clinical characteristics of the patients

Patient	Age (years)	Gender	Weight (kg)	Primary disease	Subcutaneous emphysema	Pneumothorax	Mechanical ventilation before TPM (days)	Time of diagnosis (days after admission)	The size of drainages (Fr)	Location of drainage	Time of operation(mins)
1	1	Female	12.6	Severe pneumonia	Yes	Yes	1	1	10	L2	10
2	1	Male	10	Foreign body in trachea	Yes	Yes	2	2	8	L2	14
3	11	Male	27.5	Foreign body in trachea	Yes	No	0	1	10	L2	12
4	5	Female	22	Chemotherapy	Yes	No	30	50	10	R3	15
5	3	Female	16	Severe pneumonia	Yes	Yes	10	13	10	L2	10
6	0.4	Male	7.3	Postoperative	Yes	Yes	3	4	8	L2	11
7	11	Female	38	Blunt trauma	Yes	No	1	3	10	R3	10
8	0.1	Female	3.5	Severe pneumonia	Yes	Yes	1	2	4	R2	8
9	2	Female	14	Severe pneumonia	Yes	No	56	78	10	L2	15
10	3	Female	15	Severe pneumonia	No	No	3	4	10	L3	10
11	5	Male	26	Bronchial asthma	Yes	Yes	0	5	10	R2	10
12	3	Female	16	Blunt trauma	Yes	Yes	5	5	10	R2	12
13	0.1	Male	2.7	Severe pneumonia	No	No	3	4	6	R2	8
14	7	Male	24	Severe pneumonia	No	No	1	1	10	R3	10
15	1	Male	11.5	Foreign body in trachea	Yes	Yes	0	1	10	R3	12
16	1	Male	10	Foreign body in esophagus	Yes	Yes	1	2	8	R2	15
17	3	Male	16	Bronchial asthma	Yes	No	0	10	10	L3	13
18	0.25	Female	5.5	Covid-19	No	No	1	1	6	R3	15
19	0.42	Male	8.5	Covid-19	Yes	Yes	3	3	8	L3	14
	3.1 ± 3.4	M:52.6%	15 ± 9.1		Yes:78.9%	Yes:52.6%	6.4 ± 13.8	10 ± 19.8	10 Fr:63.2%	R:52.6%	11.8 ± 2.4

TPM: tension pneumomediastinum.

## Results

### Medical history and diagnosis

A total of 19 children (10 males and 9 females) with a mean age of 3.1 ± 3.4 (0.1–11) years and weight of 15 ± 9.1 (2.7–38) kg were recruited in this study. Primary diseases: Seven patients (36.8%) had pneumonia and presented severe coughing and shortness of breath. Two patients had COVID-19, 2 patients had bronchial asthma, 4 patients had a foreign body in the trachea or oesophagus, and 2 patients had blunt chest trauma; 1 patient had a case occurring secondary to thoracoscopic segmentectomy, and 1 was receiving chemotherapy. Fifteen patients (78.9%) required ventilator support, and the other 4 patients (21.9%) required only oxygen. All patients required treatment with broad-spectrum antibiotics. All patients were diagnosed by chest CT scan; 11 additionally underwent flexible bronchoscopy, and 4 underwent oesophagoscopy. Our data showed that 52.9% of patients with tension pneumomediastinum had associated pneumothorax, and 82.4% had associated subcutaneous emphysema (Table 1).

### Management of drainage tube

The mean procedure time was 11.8 ± 2.4 min (range 8 to 15 min), the mean drainage time was 6.7 ± 3.4 days (range 2 to 13 days), and the mean hospitalization time after the operation was 12.5 ± 4.6 days. Parasternal approach drainage was performed in all patients, with 9 performed on the left and 10 on the right. Of the 19 drains, 12 were 10 Fr (63.2%), 6 were 6–8 Fr (31.6%), and only one was 4 Fr. Ten patients (52.6%) had unilateral or bilateral pneumothorax, of whom 4 underwent additional thoracentesis drainage.

### Evaluate of the efficacy of parasternal approach drainage

We recorded preoperative clinical parameters, including heart rate (HR), blood pressure (BP), blood oxygen saturation (SPO2), and fraction of inspiration O2 (FIO2), which all improved significantly after surgery. The ventilator setting decreased from the preoperative period, including positive end-expiratory pressure (PEEP) and peak inspiratory pressure (PIP). There were no severe complications, such as haemothorax, empyema, or damage to the lung parenchyma or mediastinal structure. Drainage was effective in all patients as assessed by comparing images and ventilatory parameters, and no additional surgical treatment was needed. There was no recurrence during the follow-up of more than 2 months. (Table 2)

**Table 2:** Pneumomediastinum associated symptoms and clinical outcomes

Patient	Pneumomediastinum associated symptoms	Pre-procedural abnormity clinical data	Post-procedural clinical status	Duration from drainage to symptom relief (hours)	Outcome
1	Hemodynamic instability	HR 170; BP 126/79; SPO_2_ 95%; FIO_2_ 60%; PEEP 5; PIP 22	h 110; BP 102/56; SPO_2_ 97%; FIO_2_ 40%; PEEP 4; PIP 21	4 h	Drain removal on day 13. No complication. Discharged from hospital after 16 days. No recurrence.
2	Respiratory failure	HR 110; BP 102/79; SPO_2_ 90%; FIO_2_ 80%; PEEP 5; PIP 24	h 105; BP 98/60; SPO_2_ 99%; FIO_2_ 40%; PEEP 4; PIP 21	2 h	Drain removal on day 6. No complication. Discharged from hospital after 8 days. No recurrence.
3	Hemodynamic instability	HR 162; BP 136/97; SPO_2_ 97%;	HR 98; BP 111/77; SPO_2_ 98%;	2 h	Drain removal on day 5. No complication. Discharged from hospital after 7 days. No recurrence.
4	Hemodynamic instability	HR 60; BP 73/55; SPO_2_ 97%; FIO_2_ 50%; PEEP 5; PIP 26	h 105; BP 102/73; SPO_2_ 99%; FIO_2_ 50%; PEEP 5; PIP 24	1 h	Drain removal on day 6. No complication. Discharged from hospital after 16 days. No recurrence.
5	Hemodynamic instability	HR 65; BP 77/61; SPO_2_ 98%; FIO_2_ 45%; PEEP 5; PIP 20	h 105; BP 102/73; SPO_2_ 99%; FIO_2_ 45%; PEEP 5; PIP 20	2 h	Drain removal on day 5. No complication. Discharged from hospital after 14 days. No recurrence.
6	Hemodynamic instability	HR 200; BP 99/67; SPO_2_ 99%; FIO_2_ 40%; PEEP 5; PIP 17	h 153; BP 80/55; SPO_2_ 99%; FIO_2_ 40%; PEEP 5; PIP 16	3 h	Drain removal on day 3. No complication. Discharged from hospital after 7 days. No recurrence.
7	Hemodynamic instability	HR 133; BP 138/99; SPO_2_ 96%; FIO_2_ 60%; PEEP 6; PIP 30	h 90; BP 108/79; SPO_2_ 98%; FIO_2_ 60%; PEEP 5; PIP 28	5 h	Drain removal on day 2. No complication. Discharged from hospital after 6 days. No recurrence.
8	Respiratory failure	HR 150; BP 76/50; SPO_2_ 90%; FIO_2_ 70%; PEEP 5; PIP 16	h 140; BP 79/53; SPO_2_ 99%; FIO_2_ 50%; PEEP 4; PIP 14	2 h	Drain removal on day 5. No complication. Discharged from hospital after 11 days. No recurrence.
9	Respiratory failure	HR 120; BP 89/60; SPO_2_ 87%; FIO_2_ 80%; PEEP 5; PIP 21	h 110; BP 80/53; SPO_2_ 95%; FIO_2_ 60%; PEEP 4; PIP 18	2 h	Drain removal on day 10. No complication. Discharged from hospital after 25 days. No recurrence.
10	Hemodynamic instability	HR 63; BP 60/39; SPO_2_ 95%; FIO_2_ 40%; PEEP 5; PIP 22	h 85; BP 95/63; SPO_2_ 95%; FIO_2_ 40%; PEEP 4; PIP 20	4 h	Drain removal on day 7. No complication. Discharged from hospital after 14 days. No recurrence.
11	Hemodynamic instability	HR 55; BP 58/33; SPO_2_ 97%;	HR 80; BP 98/65; SPO_2_ 98%;	2 h	Drain removal on day 4. No complication. Discharged from hospital after 11 days. No recurrence.
12	Respiratory failure	HR 88; BP 90/57; SPO_2_ 87%; FIO_2_ 75%; PEEP 5; PIP 25	h 85; BP 95/63; SPO_2_ 95%; FIO_2_ 45%; PEEP 4; PIP 22	6 h	Drain removal on day 11. No complication. Discharged from hospital after 17 days. No recurrence.
13	Hemodynamic instability	HR 205; BP 96/76; SPO_2_ 98%; FIO_2_ 45%; PEEP 5; PIP 14	h 140; BP 75/53; SPO_2_ 99%; FIO_2_ 45%; PEEP 5; PIP 14	5 h	Drain removal on day 3. No complication. Discharged from hospital after 10 days. No recurrence.
14	Hemodynamic instability	HR 139; BP 137/99; SPO_2_ 98%; FIO_2_ 40%; PEEP 5; PIP 25	h 103; BP 108/76; SPO_2_ 99%; FIO_2_ 40%; PEEP 5; PIP 24	4 h	Drain removal on day 13. No complication. Discharged from hospital after 16 days. No recurrence.
15	Hemodynamic instability	HR 170; BP 123/77 SPO_2_ 97%;	HR 125; BP 98/65; SPO_2_ 98%;	4 h	Drain removal on day 5. No complication. Discharged from hospital after 9 days. No recurrence.
16	Hemodynamic instability	HR 180; BP 99/79; SPO_2_ 95%; FIO_2_ 40%; PEEP 4; PIP 17	h 110; BP 102/56; SPO_2_ 97%; FIO_2_ 40%; PEEP 4; PIP 16	4 h	Drain removal on day 6. No complication. Discharged from hospital after 10 days. No recurrence.
17	Hemodynamic instability	HR 156; BP 139/87; SPO_2_ 97%;	HR 106; BP 102/77; SPO_2_ 98%;	3 h	Drain removal on day 4. No complication. Discharged from hospital after 12 days. No recurrence.
18	Hemodynamic instability	HR 207; BP 98/70; SPO_2_ 98%; FIO_2_ 45%; PEEP 4; PIP 15	h 207; BP 98/70; SPO_2_ 98%; FIO_2_ 45%; PEEP 4; PIP 15	4 h	Drain removal on day 8. ECMO was withdrawn on day 8. No complication. Discharged from hospital after 23 days. No recurrence.
19	Hemodynamic instability	HR 180; BP 66/44; SPO_2_ 98%; FIO_2_ 40%; PEEP 4; PIP 16	h 147; BP 87/55; SPO_2_ 99%; FIO_2_ 40%; PEEP 4; PIP 16	3 h	Drain removal on day 7. No complication. Discharged from hospital after 15 days. No recurrence.

HR:heart rate (bpm); BP: blood pressure (mmHg);SPO_2_:blood oxygen saturation; FIO_2_: fraction of inspiration O_2_; PEEP: positive end-expiratory pressure (cmH_2_O). PIP: peak inspiratory pressure.

## Discussion

### Pathophysiology of pneumomediastinum

Pneumomediastinum may occur spontaneously or as a result of other underlying conditions. Spontaneous pneumomediastinum occurs when air from ruptured alveoli travels through the pulmonary interstitium and bronchovascular sheaths towards the pulmonary hila and into the mediastinum. This phenomenon was first described by Macklin et al. in 1944 and is known as the Macklin effect [14]. Conversely, secondary pneumomediastinum is commonly associated with trauma, such as injuries to the oesophagus or bronchi. When there is a sudden increase in mediastinal pressure, the pleura in the mediastinum may rupture, resulting in pneumothorax. The air then spreads to the neck and chest through the loose subcutaneous layer, causing extensive subcutaneous pneumatosis. As a result, patients with tension pneumomediastinum often present with concurrent pneumothorax and subcutaneous emphysema. Our data show that 52.9% of patients presented with pneumothorax, and 82.4% had subcutaneous emphysema.

### Pneumomediastinum associated with COVID-19

Pneumomediastinum is a complication of COVID-19 that has been reported in children [15, 16]. The mechanisms would be alveolar injury secondary to viral infection combined with the rupture of the alveolar wall due to the increased pressure resulting from cough. Mechanical ventilation and air pressure injury are also associated with the development of pneumomediastinum [17]. COVID-19 usually presents as a clinically uncomplicated process in children. In recent data, 8% of children with severe acute respiratory distress syndrome require intensive care, only 1% of whom may require ECMO [18]. Once associated with pneumomediastinum, pneumothorax, and subcutaneous emphysema, the lengths of ICU and hospital stay increase, and the mortality rate also increases [19]. Early drainage can decrease the associated complication and mortality rates [15]. Two cases of COVID-19 causing acute respiratory distress syndrome (ARDS) and TPM have been reported. Both patients had severe ARDS with secondary pulmonary infection requiring invasive ventilation and moderate PEEP. Both required vasopressors to maintain hemodynamics and allow mediastinal drainage, and one patient received extracorporeal membrane oxygenation. Fortunately, both patients recovered and were discharged from the hospital.

### The treatment focuses on cause identification and surgical drainage

Many conditions can lead to alveolar ruptures, such as bronchial asthma, severe coughing or vomiting, and other activities associated with Valsalva modulation [14]. The main risk factor for developing pneumomediastinum in hospitalized children is mechanical ventilation, and inappropriate airway pressure may lead to alveolar rupture. Pneumomediastinum may be an ominous sign of damage to mediastinal organs, including the trachea or oesophagus, and treatment should focus on identifying the cause [20, 21]. In our cases, 4 patients had a history of foreign bodies in the trachea or oesophagus, 2 had blunt chest trauma, 1 had lobectomy, and one received chemotherapy. Treatment of patients with tension pneumomediastinum should be started before cardiac tamponade develops [5]. In our case, a patient received foreign body removal by gastroscopy. Because the symptoms of pneumomediastinum were not obvious, the patient was not diagnosed early nor examined until the blood pressure decreased on the second day after gastroscopy. We suggest that the possibility of pneumomediastinum should be considered in patients with the risk factors mentioned above. Traditional surgical approaches include tracheotomy [4], sternotomy [6], and mediastinotomy [7]. However, parasternal approach mediastinal puncture and drainage is a good option for patients at higher risk for severe respiratory and cardiovascular disease [1, 8–10].

### CT is an essential means for diagnosing and treating pneumomediastinum

CT is the gold standard for diagnosing pneumomediastinum because of its higher sensitivity and specificity than X-ray [10]. In addition to showing flattening of the anterior cardiac contour, CT scans can sometimes also show compression of the aorta, vena cava, or tracheobronchial tree [4]. CT is essential to identify the source of pneumomediastinum, thus avoiding unnecessary invasive procedures. There are few reports of mediastinal puncture guided by ultrasound and fluoroscopy [5]. The parasternal long-axis and short-axis views of the heart were difficult to ascertain due to an air artefact that seemed to coincide with respiratory variation; therefore, ultrasound is not recommended [22]. In contrast, CT imaging guidance allows for more precise localization and planning of the safest path to avoid damage to blood vessels, lung parenchyma, and other vital structures. Our patients were successfully catheterized under CT imaging guidance, and no severe complications occurred.

### Advantages of pigtail catheters: safe, minimally invasive, and effective

The pigtail catheter, known as the Fuhrman catheter [23], is a spiral, single-lumen polyurethane catheter with a size ranging from 5 to 12 Fr. Unlike traditional chest tubes, pigtail catheters are not placed via blunt chest wall dissection but through a modified Seldinger technique [11]. The technique is simple and easy to master, with an average operating time of 11.8 ± 2.4 min. A fine needle was used to puncture the mediastinum, which prevented the mediastinal organs from severe damage. The head of the catheter is automatically bent into a pigtail shape so that the stimulation to the mediastinal organs is slight. We used a suture to fix the catheter, which can effectively reduce the incidence of catheter detachment caused by traction. A catheter with the appropriate calibre is usually selected according to age, weight, and disease state. We prefer to place a small chest tube (8–14 Fr) as recommended by the Danish Pulmonary Society (DLS) [13]. In general, 6–8 Fr pigtail catheters were used for patients weighing < 10 kg, and 10 Fr catheters were used for patients weighing > 10 kg and adjusted to the situation. Of 19 drains, 12 were 10 Fr (63.2%), 6 were 6–8 Fr (31.6%) and only one was 4 Fr. It is necessary to have multiple side holes to avoid mediastinal connective tissue blocking the side holes [8]. Roberts, J. S. et al. [24] reported a 20% rate of drain complications, including drainage failure, dislocation or kinks, loss of ventilation fluid, empyema, and drain disconnections. However, no related complications were observed in our study.

### Limitations of the study

Despite the success of 19 cases, this study has many limitations. Although pneumomediastinum occurs mainly in the anterior mediastinum, the approach is inappropriate for posterior pneumomediastinum drainage. It was a single-centre, small-sample study, retrospective analysis, and lacked a control group. Therefore, more cases are needed in the future to confirm the current findings using prospective, comparative studies.

## Conclusion

CT imaging-guided parasternal approach drainage is safe and feasible for children with tension pneumomediastinum.

## Data Availability

The data supporting this study’s findings are available on request from the corresponding author. The data are not publicly available due to privacy or ethical restrictions.
